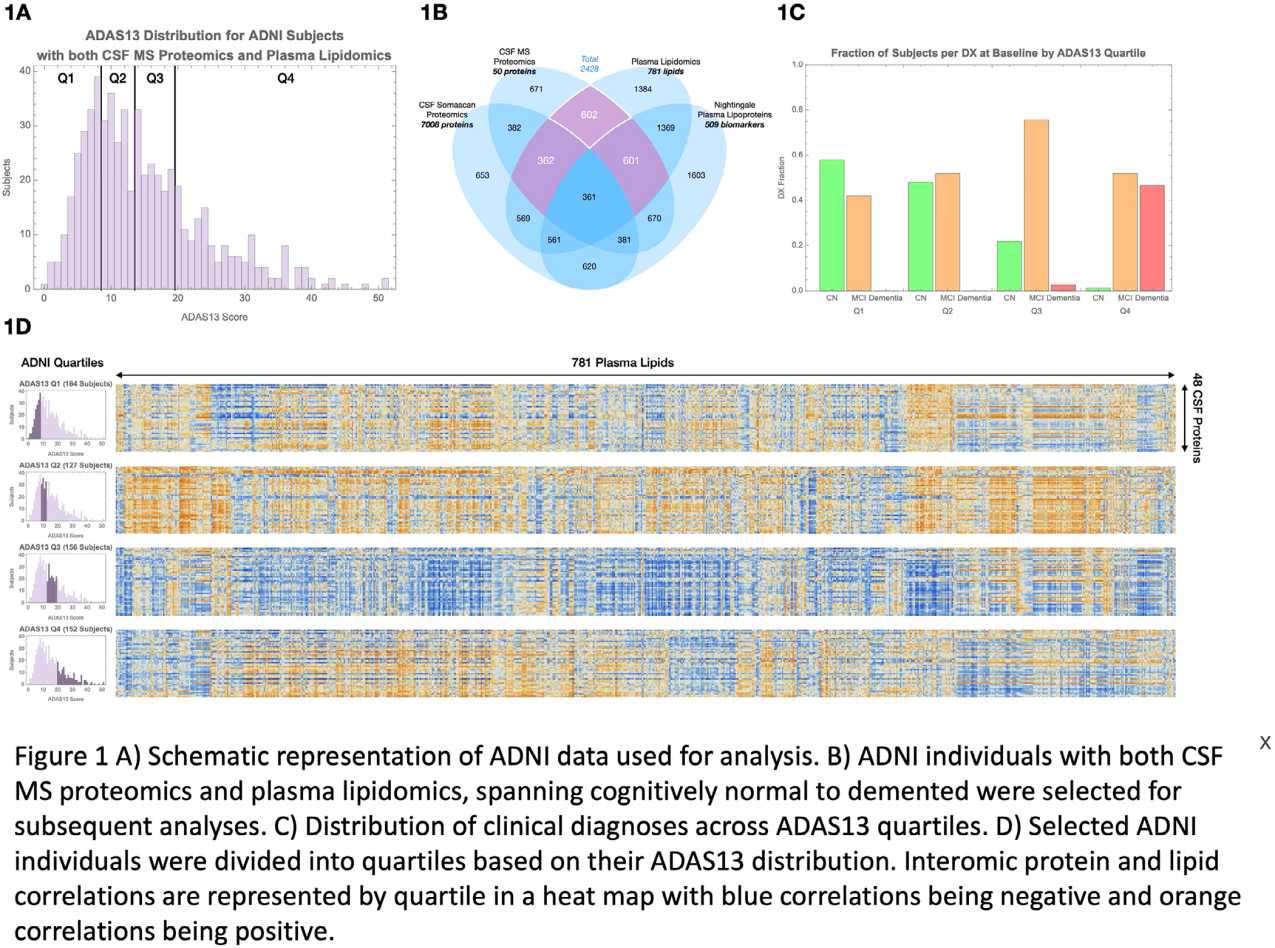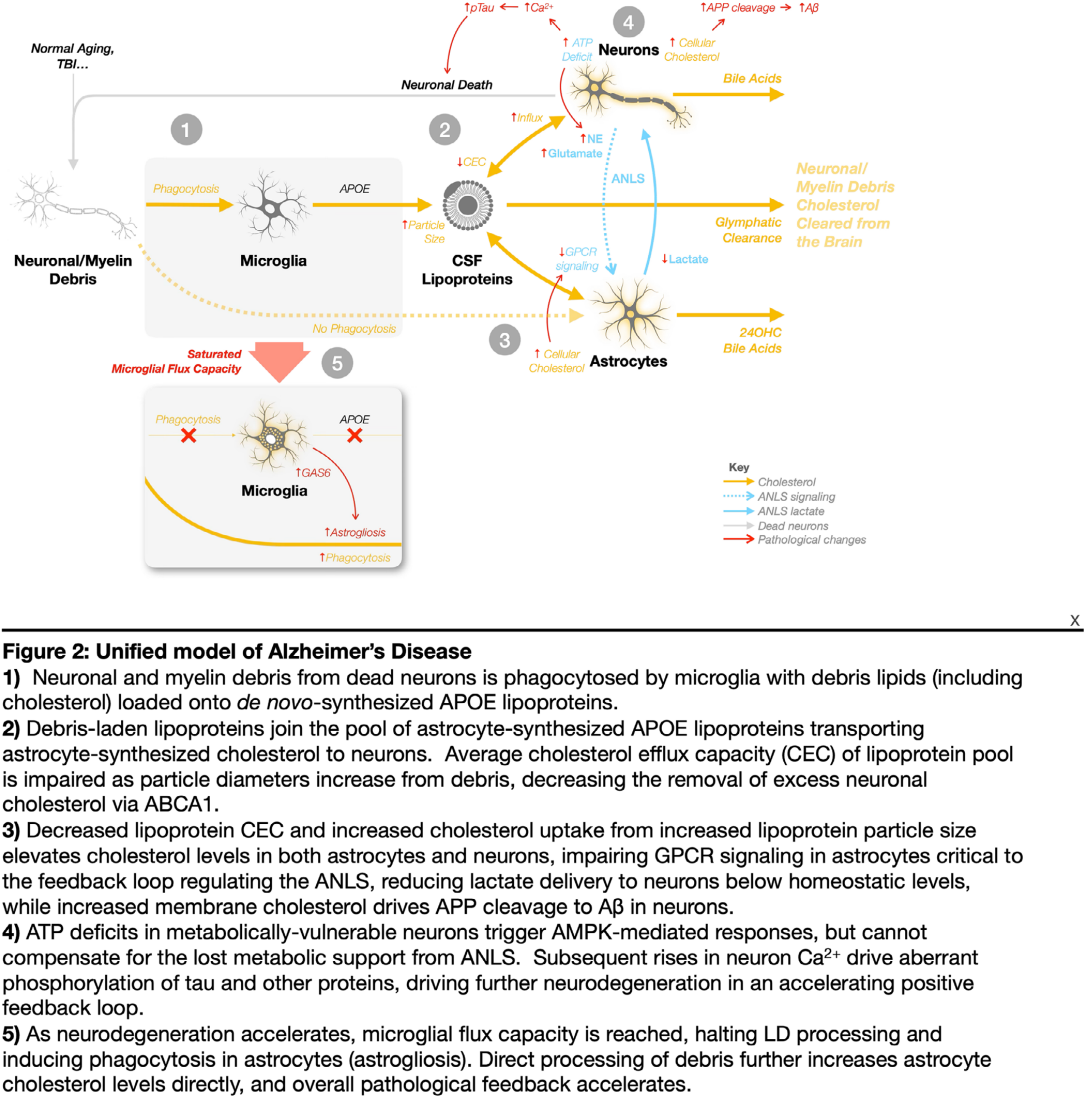# Multiomic Evidence for a Unified Model of Alzheimer's Disease Etiology Linking Astrocyte‐Neuron Lactate Shuttle Impairment and Microglial Flux Capacity

**DOI:** 10.1002/alz70855_107438

**Published:** 2025-12-24

**Authors:** Tom Paterson, Jennifer Rohrs, Timothy J. Hohman, Peter J Meikle, Rima F. Kaddurah‐Daouk, Mark Mapstone, Allan I. Levey, Leroy Hood, Cory C Funk

**Affiliations:** ^1^ Fulcrum Neuroscience, Palo Alto, CA, USA; ^2^ Vanderbilt Memory and Alzheimer's Center, Vanderbilt University School of Medicine, Nashville, TN, USA; ^3^ Baker Heart and Diabetes Institute, Melbourne, VIC, Australia; ^4^ Baker Department of Cardiometabolic Health, University of Melbourne, Melbourne, VIC, Australia; ^5^ Duke Institute for Brain Sciences, Duke University, Durham, NC, USA; ^6^ University of California, Irvine, Irvine, CA, USA; ^7^ Goizueta Alzheimer's Disease Research Center, Emory University, Atlanta, GA, USA; ^8^ Phenome Health, Seattle, WA, USA; ^9^ Buck Institute for Research on Aging, San Francisco, CA, USA; ^10^ Institute for Systems Biology, Seattle, WA, USA

## Abstract

**Background:**

Lipid biology is profoundly disrupted in AD, as evidenced by abundant genetic studies highlighting the roles of lipid metabolism, endosomal/lysosomal trafficking, bioenergetics, and other cellular and molecular alterations. Despite these observations, there is currently no unified hypothesis to explain the etiology of AD that reconciles lipid disruption with neuronal loss.

**Method:**

Using baseline measurements from ADNI plasma lipidomics and CSF proteomics, extensive reconciliation of the literature, and methods related to systems engineering and QSP, we built a model of brain homeostasis centered around feedback loops and mass balance. We then apply our model to several large multiomic datasets, including those in ADNI and GNPC for validation.

**Result:**

We identified multiomic/proteomic evidence of two convergent feedback loops driving AD pathophysiology: 1) Saturated Microglial Flux Capacity: Microglia become overwhelmed by neuronal and myelin debris, leading to impaired processing. 2) Impaired ANLS Signaling: Elevated astrocyte membrane cholesterol disrupts ANLS, compromising neuronal metabolic support.

**Conclusion:**

Neurodegeneration in AD is driven by the brain's failure to process excess cholesterol from neuronal debris, resulting in widespread lipidomic, metabolic, and proteomic disruptions. Disruptions in lipid homeostasis occur upstream of both amyloid and tau pathology, as excess cholesterol accumulation enhances APP translocation to γ‐secretase, accelerating amyloid production, while also impairing astrocytic metabolic support, leading to ATP deficits, elevated intracellular Ca²⁺, and aberrant tau phosphorylation. This breakdown in cholesterol homeostasis offers a comprehensive explanation for the observed multi‐omic alterations in AD, positioning lipid dysregulation as a primary driver of disease progression. Our unified hypothesis enables us to leverage large omics data to learn new biology, and identify and evaluate novel therapeutic targets for both the prevention and treatment of AD.